# Syllable-first rather than letter-first to improve phonemic awareness

**DOI:** 10.1038/s41598-020-79240-y

**Published:** 2020-12-17

**Authors:** Maria Vazeux, Nadège Doignon-Camus, Marie-Line Bosse, Gwendoline Mahé, Teng Guo, Daniel Zagar

**Affiliations:** 1 LISEC UR 2310, University of Strasbourg, University of Haute-Alsace, University of Lorraine, Strasbourg, Mulhouse, Nancy, France; 2 INSPÉ, Faculty of Education and Lifelong Learning, Académie de Strasbourg, France; 3 CNRS, LPNC, Univ. Grenoble Alpes, Univ. Savoie Mont Blanc, 38000 Grenoble, France; 4 Univ. Lille, CNRS, UMR 9193 - SCALab - Sciences Cognitives et Sciences Affectives, Lille, F-59000 France; 5 CNRS, ATILF, Université de Lorraine, 54000 Nancy, France

**Keywords:** Psychology, Human behaviour

## Abstract

The present study investigates the nature of the spelling-to-sound correspondences taught to enhance phonemic awareness in prereaders. The main assumption in the literature is that learning the alphabetic code through letter-to-phoneme correspondences is the best way to improve phonemic awareness. The alternative syllabic bridge hypothesis, based on the saliency and early availability of syllables, assumes that learning to associate letters to phonological syllables enables phoneme units to be the mirror of the letters and to become accessible, thereby developing phonemic awareness of prereaders. A total of 222 French-speaking prereaders took part in a 4-session learning program based on correspondences either between letters and syllables (letters-to-syllable group) or between letters and phonemes (letter-to-phoneme group), and the fifth last session on coding and decoding. Our results showed a greater increase in phonemic awareness in the letters-to-syllable group than in the letter-to-phoneme group. The present study suggests that teaching prereaders letters-to-syllable correspondences is a key to successful reading.

Learning to read is considered to be a code-cracking action^1^ as beginning readers learn the associations between letters and sounds. For each word, children have to associate letters and their sounds, to concatenate them to produce the phonological form of the word, and then to relate it to its meaning^2^. Before receiving reading instruction focused on letter-to-sound correspondences, prereaders gain knowledge and experience about spoken and written language^3,4^. Two specific abilities of prereaders have been reported to correlate with their future reading level while controlling for other environmental or genetic parameters: IQ, vocabulary level, mother’s education level or socio-economic backgrounds^5,6^: (i) letter knowledge^7^ and (ii) phonological awareness (see^8^ for a review).

Letter knowledge refers to the name of a letter and its sound. A large set of research suggested that letter-name knowledge appears before letter-sound knowledge^9,10^ but this may be true only when letter-name is taught before letter-sound^11^. Letter knowledge is a good predictor of reading skills^12–18^. Learning and memorizing letter names seems to be useful to learn the alphabetic code^19^, especially when letter names contain letter sounds^20–24^. Recently, Lerner and Lonigan^25^ reported that the initial level of letter-name knowledge of prereaders predicted growth in phonological awareness, and that the initial level of phonological awareness of prereaders predicted growth in letter-name knowledge, indicating that both skills are bi-directionally related.

Phonological awareness refers to the intentional ability to manipulate units of speech^26^ and develops before and during learning to read. Phonological awareness is a single cognitive capacity measured by different tasks that prereaders and beginning readers perform in the following order: detect a sound, blend sounds, and then elide a sound^27–29^. The size of the speech units to which the tasks relate also follows a progression from large-to-small units (word, syllable, onset-rime, phoneme^30–32^). As noted above, there is a large literature suggesting that phonological awareness relates to reading skills^33–41^. In addition, the phonological awareness level predicts the left lateralization of the N170 component^42^, which reflects the neuronal specialization of the visual word form area and visual expertise for print processing^43–45^. More precisely, phonemic awareness—the endpoint of phonological awareness because it reflects the capacity of organization of the finest level of phonology—is the strongest longitudinal predictor of reading and spelling skills^12,13,46–50^. In alphabetic scripts, children must gain access to phonemes to use the letters^51^. Hulme et al.^52^ (p. 362), argued that “phoneme awareness can be considered as a marker of the status of underlying phonological representations and their readiness to act as a foundation for the development of orthographic knowledge”. The mastery of phonemic skills helps beginning readers to organize and master the alphabetic code in order to spell and read^53,54^. Thus, phonemic awareness cannot only be considered as a predictor of literacy in the view of causality^55^, but also as a marker of the mastery of the alphabetic code in the learning-to-read process^12,19,52^. Phonemic awareness performances can then be used to capture the very first signs of alphabetical code acquisition.

Training phonological awareness before learning to read results in gains in reading, and learning to read in turn results in gains in phonemic awareness^56–64^. Evidence comes from studies with beginning readers^53,65–70^, illiterate adults^71–74^, disabled adults^75^, adults learning to read^76^ or poor adult readers^77^ who were unable to manipulate phonemes, suggesting that phonological awareness skills are the result of reading acquisition^78^. Therefore, experience with alphabetic systems refines phonological awareness^79–81^, and the ability to intentionally manipulate phonemes develops with phonics-based reading instruction ^8,78,82–87^.

Phonics-based reading instruction refers to “the teaching of correspondences between letters or groups of letters and their pronunciations”^51^ (p. 50). Among these correspondences, the question of the precise nature of grapho-phonological relationships that enhance phonemic awareness remains open. In the larger framework of learning to read theories, the dominant hypothesis postulates that learning correspondences between elementary units of language, that is, graphemes and phonemes, is the way to learn the alphabetic code^88–91^. The assumption derived from these models is that learning grapheme-phoneme associations is the way to develop phonemic awareness, as a corollary of our writing alphabetic system^8^. Several studies have shown that phonics instruction based on grapheme-phoneme correspondences improves the development of phonemic awareness^92–101^.

However, the grapheme-phoneme correspondences as enhancing phonemic skills compared to other forms of instruction has never been proved to be the most effective^52^. Moreover, the effectiveness of these correspondences for starting literacy is reported to be inconsistent^102^. Here we defend an alternative hypothesis concerning precise phonics-based reading instruction that enhances phoneme awareness. We assume that teaching letters-to-syllable connections is more efficient in boosting phoneme awareness than grapheme-phoneme connections and in developing thereafter alphabetic code acquisition. Our assumption, which refers to the syllabic bridge hypothesis^103^ stems from the accessibility and awareness of syllable units^68,104,105^, long before any experience of written language^106^. The syllable is a natural unit of speech production and more readily perceptible than a phoneme in a speech stream^107–110^. Syllable awareness is already present in 3–4 year old children^26^ whereas phoneme awareness does not appear before the age of six. The syllable is thus the earliest available phonological unit^32^ and appears prior to literacy. Syllables also play a role in the process of visual word recognition when learning to read, as beginning readers activate phonological syllables from written word perception (with a word-spotting paradigm^111^; in a French lexical decision task^112^; in a target detection task^113^; in an illusory conjunction task^114^; in a Spanish lexical decision task^115^). Gallet et al.^116^ tested an early reading intervention based on the syllabic bridge hypothesis in French elementary schools and showed progress in written-word identification (in Finnish for syllable training^117,118^). In addition to being easily accessible units early in both speech and written language, syllables could be functional units in the process of learning to read and in the development of phonemic awareness.

The syllabic bridge hypothesis derived from the Developmental Interactive Activation Model with Syllables (DIAMS model^103^). The major stage in this reading acquisition model is the building of a set of connections between phonological syllable units and letters, allowing children to grasp the alphabetic principle. The previously acquired bundle of syllabic connections is the knowledge base from which phonemes become accessible and conscious units. For example, having learned that the letters “pa” correspond to the phonological syllable /pa/, children are able to allocate attention to shorter components of both printed and sound language, and to progressively understand that there are two visible letters in “pa” that map to two different parts of the syllable, /p/ and /a/. In other words, with phonics-based reading instruction on letters-to-syllables connections, beginning readers progressively develop phoneme awareness on the basis of written syllable knowledge, and thereafter establish detailed connections between letters and phonemes (i.e., acquire the alphabetic code). The first piece of evidence of the easy construction of the syllabic bridge was reported by Doignon-Camus and Zagar^103^, in which the bundle of syllabic connections was built after only a few minutes of learning letters-syllable correspondences (e.g., “pi” is pronounced /pi/) in 5-year-old prereaders. Here, we focused on the strong assumption of the syllabic bridge, by directly testing whether building the syllabic bridge between letters and phonological syllables leads to phoneme awareness.

In a recent study with Brazilian prereaders^119^, the question of the nature of grapho-phonological relationships that enhance phonemic awareness has apparently been adressed. In that study, Brazilian Portuguese-speaking prereaders received instruction in grapho-phonological relationships but also phonological awareness training. One group received letter-to-phoneme instruction plus phonemic awareness training, and the other group received letters-to-syllable instruction plus syllable awareness training. Pre-readers performed better in reading, spelling and phonemic segmentation after benefiting from phoneme-based rather than syllable-based training. Contrary to the above study by Sargiani et al.^119^, the present research disentangles the influence of spelling-to-sound instructions and phonological awareness training to develop phonemic awareness and focuses solely on the nature of spelling-to-sound correspondences to be learned that enhance the development of phonemic awareness, by comparing the efficiency of learning letters-to-syllable correspondences (syllabic bridge hypothesis) and learning letter-to-phoneme correspondences (dominant hypothesis). To this end, we implemented a controlled, longitudinal teaching program in non-reading, French-speaking children attending preschool. They received four sessions of instruction based on correspondences either between letters and syllables (letters-to-syllable group) or between letters and phonemes (letter-to-phoneme group). All the children had an introductory session on coding and decoding. Phonemic awareness skills were assessed at three time-points (T1, T2, T3) with a final phoneme elision task. Phoneme elision is widely used in the literature, with illiterates, prereaders and beginning readers. Among other phonological awareness tasks, it has high reliability^16^ and is the strongest predictor of variations in reading skills in children^17,120,121^. Final phoneme elision task appears to be the most appropriate task for assessing phonological awareness skills^120^ and a reliable screening tool for reading abilities^122^. As the French language has great consistency^123^ and saliency^124^ of syllables, we assume that teaching the mapping between letters and syllable enhances phonemic awareness in French prereaders^103^.

## Results

The aim of the first analysis was to test the progression of children’s skills in reading syllables between T1 and T3. The second analysis aimed to test our hypothesis by testing the progression of phonemic awareness skills in each group. The third analysis was conducted on the progression of phonemic awareness skills as a function of children’s initial skills at T1.

### Progression of syllable reading

**Figure 1:**
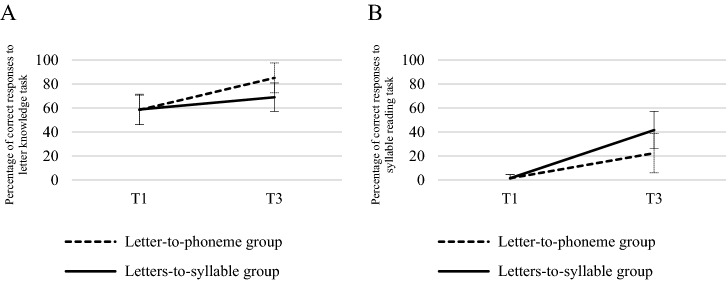
Percentage of correct responses for the two groups at T1 and T3 in letter name and sound knowledge (**A**) and syllable reading tasks (**B**). Notes. Panel A : letter-name task: letter-to-phoneme group, T1: 74%; T3: 87%; letters-to-syllables group T1: 74%; T3: 81%. Letter-sound task: letter-to-phoneme group, T1: 26%; T3: 80%; letters-to-syllable group, T1: 27%; T3: 44%.

All children progress significantly between T1 and T3 on syllable reading (*F*(1,220) = 212.79, *p* < .0001). Moreover, syllable reading performance (Fig. 1B) increased between T1 and T3 more in the letters-to-syllable group (1.4% vs. 41.6% of correct responses) than in the letter-to-phoneme group (1.4–22.3% of correct responses; *F*(1,220) = 21.25, *p* < .0001. However, this result cannot be taken as a solid evidence of the better efficiency of the letters-to-syllable training over the letter-to-phoneme training as the letters-to-syllable group was specifically trained to read syllables in the first four sessions. Then these data should simply show the efficiency of the first four teaching sessions. Similarly, knowledge of letter names and letter sounds increased between T1 and T3 more in the letter-to-phoneme group (58.4% vs. 85.1% of correct responses) than in the letters-to-syllable teaching group (58.8% vs. 68.9%; *F*(1,220) = 60.21, *p* < .0001, Fig. 1A).

### Development of phonemic awareness

**Figure 2:**
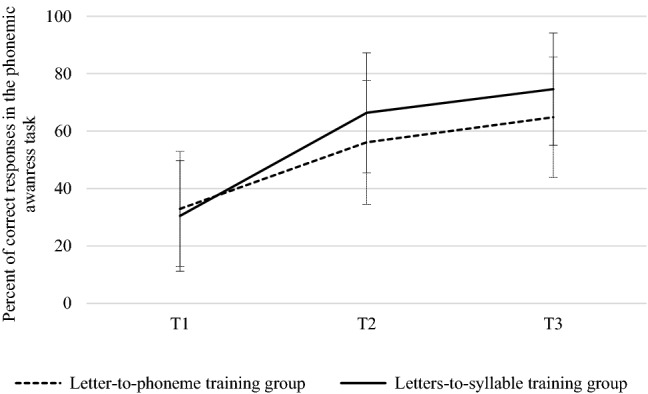
Percentage of correct responses in the final phoneme elision task at T1, T2 and T3 for both groups.

The main effect of time (T1, T2, T3) was significant on phoneme awareness scores, (*F*(2,440) = 162.18, *p* < .0001), but not the main effect of group, (*F*(1,220) = 1.48, *p* = .22). The main result was in agreement with our main hypothesis, phoneme awareness skills increased more in the letters-to-syllable group (T1: 30.4%; T2: 66.3%; T3: 74.6% of accuracy) than in the letter-to-phoneme group (T1: 32.9%; T2: 56%; T3: 64.8% of accuracy), as shown in Fig. 2 the Time x Group interaction was significant, *F*(2,440) = 5.31, *p* = .005. Planned comparisons (Bonferroni corrected) revealed a significant interaction between group and time when T1 and T2 were selected (*F*(1,220) = 6.72, *p* = .01), but not with T2 and T3 (*F*(1,220) = 0.02, *p* = .87).

A second concern was whether children were making progress on the final phoneme elision task only on items on which they had received instruction (i.e., learned syllables), or whether they transferred their skills to other items (i.e., syllables that were not learned and new syllables, including new letters), see Table 1. No significant interaction was found between group, time and syllable items, *F*(4,880) = 0.92, *p* = .44). The progress in phoneme awareness in the letters-to-syllable group compared to the letter-to-phoneme group was significant for the learned syllables (*F*(1,220) = 7.29, *p* = .007) and for new syllables (*F*(1,220) = 7.30, *p* = .007), and marginal for syllables that were not learned (*F*(1,220) = 3.60, *p* = .05).

**Table 1:** Percentage of correct responses in the final phoneme elision task for both teaching groups, according to time and type of syllables.

Group	Time	Learned syllables (%)	Not learned syllables (%)	New syllables (%)
Letter-to-phoneme	T1	32.4	33.3	32.9
	T2	57.4	55.6	55.1
	T3	67.7	64.5	62.1
Letters-to-syllable	T1	29.2	32.1	30
	T2	67.1	64.2	67
	T3	77.1	73.3	73.5

### Further investigation of developmental phonemic awareness as a function of the child’s initial knowledge

**Figure 3:**
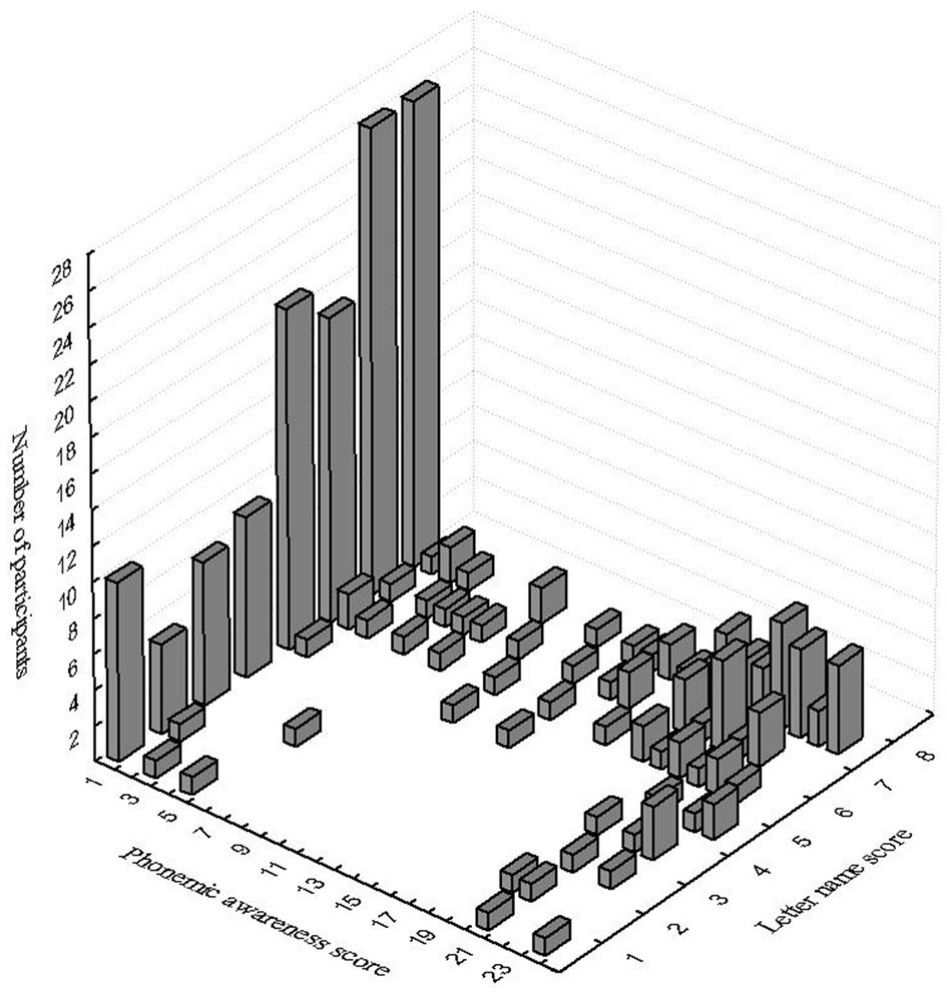
Distribution of scores of the 4 sub-groups at T1 in the letter-name knowledge and phoneme awareness tasks.

While the effect of training on phonemic awareness appeared to be greatest in the group of children who were taught letters-to-syllable correspondences, we also explored whether the effectiveness of the training was influenced by the children’s initial knowledge. As there was marked inter-individual variability in the measurements made at T1 (see Fig. 3), we divided the cohort of 222 children for analysis into four subgroups based on their initial level of phonemic awareness and letter-name knowledge (i.e., the two most important predictors of future reading ability). Children were classified as having good skills if their score was above average and as having poor skills if their score was below average (Table 2).

**Table 2:** Characteristics of the participants per subgroups and training program.

Subgroups	Low phonemic awareness / Low letter name knowledge (L–L groups) *n* = 36		Low phonemic awareness / High letter name knowledge (L–H groups)*n* = 110		High phonemic awareness / Low letter name knowledge (H–L groups) *n* = 11		High phonemic awareness / High letter name knowledge (H–H groups) *n* = 65	
Training program	Syllable *n* = 19	Phoneme *n* = 17	Syllable *n* = 61	Phoneme *n* = 49	Syllable *n* = 5	Phoneme *n* = 6	Syllable *n* = 35	Phoneme *n* = 30
Age (*M*) in months	63	61	63	63	67	64	65	66

**Figure 4:**
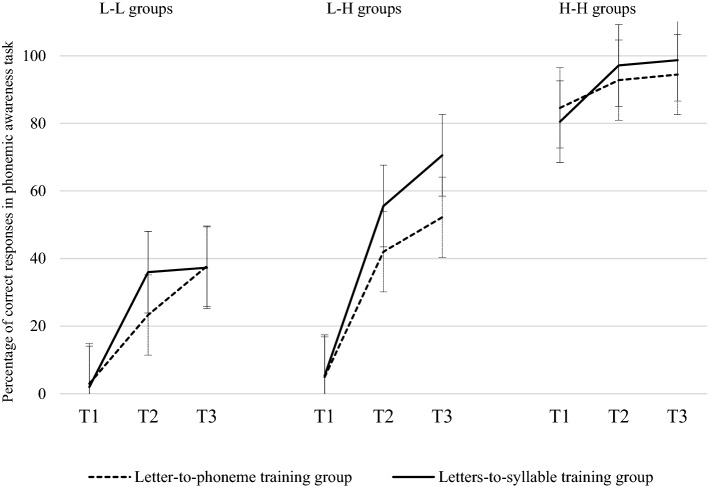
Percentage of correct responses in the final phoneme elision task at T1, T2 and T3 for both groups. *Note*. L–L groups = low phonemic awareness/low letter name knowledge. L–H groups = low phonemic awareness/high letter name knowledge. H–H groups = high phonemic awareness/high letter name knowledge.

As shown in Fig. 4 for the subgroup with low phonemic awareness and low letter name knowledge (L-L groups), the progress in phonemic awareness was similar in the two training programs, (T1: 1.9%; T2: 35.9%; T3: 37.2% for the letters-to-syllable group; T1: 2.9%; T2 : 23.2%; T3: 37.7% of accuracy for the letter-to-phoneme group). The analysis of variance between time (T1, T2 and T3) and groups was not significant, *F*(2,68) = 0.91, *p* = .40). Planned comparisons (Bonferroni corrected) reported a non-significant interaction between group and time when T1 and T2 were selected (*F*(1,34) = 1.27, *p* = .26) and a marginal interaction with T2 and T3 were selected (*F*(1,34) = 3.74, *p* = .06).

The subgroup with low phonemic awareness but high letter name knowledge (L–H groups) clearly made more progress in phoneme awareness accuracy in the letters-to-syllable group (T1: 5.3%; T2: 55.5%; T3: 70.5%) than in the letter-to-phoneme group (T1: 4.9%; T2: 42%; T3: 52.2%). Analysis of variance between time (T1, T2 and T3) and group was significant, *F*(2,216) = 3.69, *p* = .02. Planned comparisons (Bonferroni corrected) revealed a non-significant interaction between group and time when T1 and T2 were selected (*F*(1,108) = 2.80, *p* = .09), and when T2 and T3 were selected, (*F*(1,108) = 1.08, *p* = .30).

We did not analyze the subgroup with initial high phoneme awareness and low letter name knowledge because there were only 11 participants in this subgroup (*n* = 11).

Finally, in the subgroup with initial high phoneme awareness and high letter name knowledge (H–H groups), the letters-to-syllable group made more progress in phoneme awareness accuracy (T1: 80.4%; T2: 97.1%; T3: 98.6%) than the letter-to-phoneme group (T1: 84.5%; T2: 92.7%; T3: 94.4%), as revealed by the significant Time (T1, T2, T3) x Group interaction, (*F*(2,126) = 6.98, *p* = .001). Planned comparisons (Bonferroni corrected) revealed a significant interaction between group and time when T1 and T2 were selected (*F*(1,63) = 7.29, *p* = 0.008), but not when T2 and T3 were selected, *F*(1,63) = 0.007, *p* = .92.

## Discussion

Previous studies showed that phonemic awareness develops with reading instructions on print-to-speech mapping. The present study used phonemic awareness to capture the very first signs of the mastery of the alphabetic code. It aimed to test whether the development of phonemic awareness—as a marker of the alphabetic code acquisition—was enhanced when phonics instruction focused on letters-to-syllable or on letter-to-phoneme correspondences. The first analysis showed that the letters-to-syllable group progress better in syllable reading than the letter-to-phoneme group between T1 and T3. These data have been expected as we trained the letters-to-syllable group to read syllables during the teaching program. Our main result was the interaction between time and teaching group: the teaching of letters-to-syllable relationships was more conducive to the development of phonemic awareness than that of letter-to-phoneme relationships. More precisely, the greater progress made by the letters-to-syllable teaching group than by the letter-to-phoneme group was observed between T1 and T2, but not between T2 and T3. This result is evidence that the greater progress in phonemic awareness was due to learning letters-to-sound correspondences mediated by syllable units. Thus, the success of this learning was due to its associative nature and not to explicit instructions concerning the alphabetic principle. In contrast, a single introduction session to coding and decoding did not boost progression in phonemic awareness, nor did it alter it, since the advantage of the syllable teaching group was maintained at T3. However, we cannot exclude the possibility that this was due to the brevity of the coding and decoding session.

The main difficulty facing children who are learning to read is that, independent of each other, phonemes have no physical reality. That is why teaching methods promote learning of correspondences directly between the letter and the phoneme. Contrary to the dominant opinion that the attentional focus must be directed towards phonemes and their relations to letters, here we show that focusing on syllable units is more effective in developing phonemic awareness, as proposed in the syllabic bridge hypothesis^103^. As noted above, syllables are the easiest and earliest units to perceive in spoken language and to manipulate for prereaders. Our concept is the following: by teaching the relations between letters and syllables, children learn the pronunciation of letters depending on their context, and learn that a spoken syllable corresponds to a sequence of letters; this implicit and associative learning allows children to extract regularities between letters and phonemes. Then they can build phoneme representations in the mirror of letter representations.

This striking result has important implications for enhancing the process of learning to read. Phoneme awareness is the ability the most strongly correlated with reading acquisition. Its facilitative effect is strongest during the period in which children learn to crack the alphabetic code^8^. Phonemic awareness is thus considered as a marker of the alphabetic code acquisition in the learning-to-read process^12,19,52^. The present study clearly shows that automatic connections between letters and syllables boost the acquisition of phonemic awareness and therefore mediate successful reading acquisition. It is important to note that the children were not previously trained in phonemic awareness, thus proving that the teaching of simple relationships between letters and syllables leads to progress in phonemic awareness skills.

The complementary analysis of the data concerning the children’s initial skills has two major interests. On the one hand, it describes the population of prereaders according to the two factors that influence the development of skilled reading. The majority of prereaders (49.5%) had good knowledge of letter names, but low phonemic awareness skills and 29.2% already had both skills. In contrast, 16.2% had not yet developed either of the skills, and only 4.9% had high performances in phoneme awareness but low knowledge of letter name. Even if phonemic awareness and letter name knowledge influence one another as they develop^7,25^, our analysis of 222 prereaders clearly showed that they develop more skills in letter name than in phonemic awareness before formal reading instruction begins. This analysis leads us to conclude that there is a developmental progression of knowledge before learning to read: a) children do not present any phoneme awareness and letter name skills; b) children begin to learn the letter names; c) then children begin to develop phoneme awareness.

On the other hand, the complementary analysis provides information on how prereaders learn from the instructions they receive. The prereaders, for whom teaching the letters-to-syllable relationships was more conducive to phonemic awareness progression than that of letter-to-phoneme relationships, were prereaders with high initial knowledge of letter names (with low or high phonemic awareness, representing three-quarters of the sample). In contrast, no difference was observed between the two types of teaching on the development of phonemic awareness in prereaders with limited knowledge of letter names. This result leads us to assume that knowledge of letter names is a prerequisite for benefitting from phonics instruction based on syllable units. As Adams^51^ recalls, new knowledge is built on existing knowledge. Hence, children first develop knowledge of letter names as they “provide a more accessible link between print and speech than do letter sounds” ^7^ p. 595, and thereafter they can learn to associate letters with available phonological syllable units and consequently develop their phoneme awareness.

In conclusion, our results shed doubt on the widely accepted idea that children need to learn the correspondences between letters and phonemes in order to develop phoneme awareness. Instead, children should learn the correspondences between letters and the larger accessible phonological units, syllables. However, the acquisition of a syllabary is clearly not envisaged. Rather, we propose that after having acquired a basic knowledge of letter names, prereaders could benefit from learning the letters-to-syllable relationships that make sense given their prior knowledge and skills. From a bundle of letters-to-syllable connections, prereaders could build phoneme representations in mirror of letters, and thereafter acquire and master the alphabetic code.

## Method

### Participants

A total of 415 children attending public preschool participated in our study, and 193 were excluded. First, 65 children were excluded because none of the parents at home spoke French; then, 46 children were excluded because they were already readers at T1; finally, 82 children were excluded because they were absent from at least one teaching session or one of the three tests. The final sample comprised 222 French children (of which 136 girls), aged 5 years and 4 months on average (Table 3), from 15 different preschools. More precisely, 160 children attended in 11 schools with medium to high socioeconomic status and 62 children attended in 4 schools with low socioeconomic status, equally distributed between the two groups. They all had normal vision and hearing and presented no language disorders. Participants in both groups were matched using three literacy scores at T1 (Table 4). Child’s parents or legal guardians provided informed consent prior to inclusion in the study in accordance with the Declaration of Helsinki. This study was approved by the ethics committee of the Grenoble-Alpes University (No. IRB00010290-2017–12-12–34) and by the Director of departmental services of the Ministry of National Education (“DASEN”). All methods were performed in accordance with the aforementioned relevant guidelines and regulations.

**Table 3:** Characteristics of participants.

	Letters-to-syllable teaching	Letter-to-phoneme teaching
*n*	120	102
**Gender**		
Boys	58	46
Girls	62	56
**Lateralization**		
Right-handed	95	89
Left-handed	25	13
*M* years, months (*SD*)	5.3 (0,3)	5.3 (0.3)
Range	4.9 to 6.1	4.8 to 6

**Table 4:** Scores at the T1.

	Letters-to-syllable teaching	Letter-to-phoneme teaching	Group difference
	*M* (*SD*)	*M* (*SD*)	
Knowledge about letters name (max 8 items)	5.9 (2.1)	5.9 (2.1)	*t*(220) = .11, *p* = .9
Phonemic awareness (max 24 items)	7.3 (9.2)	7.9 (9.6)	*t*(220) = − .46, *p* = .64
Syllable reading (max 16 items)	0.2 (0.9)	0.2 (0.9)	*t*(220) = − .45, *p* = .65

### Procedure and material

**Figure 5:**
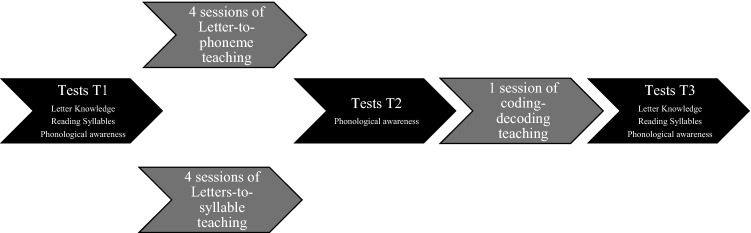
Experience design.

The design of the experiment (Fig. 5) was the following: T1 (i.e., pre-test)—teaching (four sessions)—T2 (i.e., post-test 1)—coding-decoding introduction (one session)—T3 (i.e., post-test 2). All characters (syllables and letters) were printed in capitals Calibri 72, using black type on a sheet of white A4 paper. The same typography was used for the material used for the teaching sessions and the tests.

#### Learning program

Children in both the letters-to-syllable group and letter-to-phoneme group participated in four sessions of associative learning and one introductory session to coding and decoding. Each session lasted 25 min (the usual duration of learning activities in French kindergartens) distributed over a period of 4 weeks (total learning time 125 min) in order to enhance learning^125^. Each session was conducted in small groups of 4–5 children. The letter-to-phoneme group followed a control program built with language exercises usually taught in French preschools based on learning of correspondences between a grapheme and a phoneme. Four consonants (C) with only one phonemic identity in onset position in French and 4 vowels (V) were chosen (“b”, “f”, “t”, “s”, “a”, “i”, “o”, “u”). The letters-to-syllable group followed a program centered on letters-to-syllable learning. Syllables were constructed with the same consonants and vowels as in the letter-to-phoneme group; all the syllables were CV structure, which is the most frequent in French. In a Latin-square design and in order to avoid effects of syllable material, two subsets of 8 syllables were created (“ba”, “bi”, “fa”, “fi”, “so”, “su”, “to”, “tu” and “bo”, “bu”, “fo”, “fu”, “sa”, “si”, “ta”, “ti”); randomly, half the children in the letters-to-syllable group learned set 1, and the other half learned set 2 (see S1 Supplementary information).

##### Teaching sessions

The aim was to teach children correspondences between written and spoken syllables (in letters-to-syllable group) and between letters and the corresponding name and sound (in the letter-to-phoneme group). In the first four sessions of the program, the children learned a total of 8 syllables or 8 letters depending on which group they were in; each syllable or letter being taught in at least two sessions. Five exercises were proposed during the sessions: reading, letter-phoneme matching (only in the letter-to-phoneme group), dice, lotto and relay exercises. In the reading exercise, children were asked to read three times; 1) a syllable or a letter that was presented visually while the instructor read the syllable or give the name and the sound of the letter, the children then had to repeat the syllable or the letter name and sound all together, and so on, for the three other syllables or letters; 2) the children had to read the four syllables aloud together or give the name and sound of the four letters, without the instructor; 3) each individual child had to read one of the four syllables or give the name and sound of the four letters. The letter-phoneme exercise was only added in the letter-to-phoneme group (in the two first sessions of the program) so that the duration of the teaching program was the same in the two groups. In this exercise, children had to find the first letter in the name of four pictures (e.g., the letter “s” in the picture of the “soleil”, sun in English). In the dice exercise, children had to read the syllable aloud or give the name of the letter and the sound of the syllable or the letter written on the upper side of a rolled dice; when the child gave the correct response, he/she moved his pawn forward on a 3-square board. In the lotto exercise, each child received a lotto grid with four written syllables or four letters; they had to listen to the sound of the syllable or the name of the letter pronounced by the instructor and locate the corresponding written syllable or letter on the lotto grid. In the relay exercise, which was played by a team comprising two children, the first child had to read a written-syllable aloud or produce a letter name and sound; the second child had to listen and memorize the phonological syllable or letter name and sound just heard, cross the classroom and, in a set of four written syllables or four letters, find the matching syllable or letter card, and check back with the first child; they then changed roles.

The fifth session of the teaching program was an introductory session on coding and decoding and was the same in both groups. The coding exercise corresponds to what Castles et al. (2018) call the analytic phonic programs, which “begins with whole words, and [for which] grapheme-phoneme correspondences are taught by breaking those words down into their component parts.” (p. 13): children had to choose the individual letters that corresponded to the syllable pronounced by the instructor (e.g., the instructor said /bo/ and the instruction given to children was to find the letters that correspond to the syllable; in case of failure, the correct answer was immediately given). The decoding exercise corresponds to synthetic phonic programs which “teach grapheme-phoneme correspondences individually and in a specified sequence, and children are taught early to blend (synthesize, hence the term synthetic) individual phonemes together to make words” (Castles et al., 2018, p. 13): children had to pronounce the syllable composed by the individual letters chosen by the instructor (e.g., the instructor gave the letter “b”, which sounds /b/ and the letter “o”, and the instruction given to children was to find the correct pronunciation of the two letters put together; in case of failure, the correct answer was immediately given). To respect the order of the teaching program, the two exercises were not presented to the two groups in the same order. As the letter-to-phoneme group focused on letters and phonemes, they started with the coding exercise in which they had to combine two letters. In contrast, as the letters-to-syllable focused on letters and phonological syllables, they started with the decoding exercise in which they had to break the syllable into phonemes and letters.

#### Tests

Three measures of early literacy were used: letter knowledge, phonological awareness, and syllable reading. All three tasks were measured at T1 and T3, whereas only phonological awareness was measured at T2.

##### Task 1: Letter knowledge

We used the same material as that used in the teaching sessions (i.e., “a”, “i”, “o” and “u”; “b”, “t”, “f” and “s”). Children had to say the name of each vowel and consonant and the sound of the consonants aloud. No feedback and no stopping rule when children failed were given.

##### Task 2: Final phoneme elision

The examiners presented a stimulus syllable with a CVC structure verbally and asked the children to produce the target syllable by repeating it without the final phoneme (e.g., /bak/ =  > /ba/). The examiner provided one example and two trials with feedback before starting the test. During the test itself, no feedback was given. Among the 24 items, eight were syllables learned in the teaching session (i.e., set 1 or set 2), eight were syllables that had not been learned (i.e., set 2 for participants who learned set 1; set 1 for participants who learned set 2), and eight were new syllables composed of new consonants that had not been learned in the program, such as “v” /v/, “p” /p/, “m” /m/, “r” /R/ (e.g., “vip” /vip/, “mul” /myl/). A stopping rule was applied after four incorrect items for learned syllables, and after two incorrect items for unlearned syllables and new syllables.

##### Task 3: Syllable reading

Children were asked to read 16 CV syllables, eight learned syllables and eight unlearned syllables aloud. No feedback and no stopping rule were given.

Table 3 details the program and lists the material used in each group (i.e., letters-to-syllable teaching program and letter-to-phoneme teaching program).

### Statistical analysis

All analyses were performed using STATISTICA software. Although the repeated measures were not normally distributed, it is generally accepted that ANOVA is a robust test, particularly when the sample is large enough and the groups are about the same size^126–128^. An ANOVA with group (letters-to-syllable vs. letter-to-phoneme) as a between-subject variable, time (T1, T2 and T3) and syllables (learned, not learned and novel syllables) as within-subject variable was conducted on measures of phoneme awareness. In the case of planned comparisons, the Bonferroni method was used and the significance level has been adjusted (to 0.05/2 = 0.025). ANOVAs with group and time (only T1 and T3) were conducted on letter knowledge and syllable reading scores. The datasets generated during and analysed during the current study are available in the Open Science Framework repository, https://mfr.osf.io/render?url=https%3A%2F%2Fosf.io%2Febpft%2Fdownload.

## Supplementary Information

Supplementary Information

## Data Availability

Data can be made available upon request to authors.
