# Supplementary material for: Syllable-first rather than letter-first to improve phonemic awareness
Source: Sci Rep. 2020 Dec 17;10:22130. doi: 10.1038/s41598-020-79240-y (PMC7747708; doi:10.1038/s41598-020-79240-y)
Supplement: Supplementary file 1 — Supplementary Information [file 41598_2020_79240_MOESM1_ESM.docx]

Syllable-first rather than letter-first to improve phonemic awareness

Maria Vazeux, Nadège Doignon-Camus, Marie-Line Bosse, Gwendoline Mahé, Teng Guo & Daniel Zagar

**Supplementary information**

*Tasks and material used in both training programs*

|  | Letters-to-syllable teaching program | Letter-to-phoneme teaching program |
| --- | --- | --- |
| Tests at T1 | Letter knowledge, phonemic awareness and syllable reading | Letter knowledge, phonemic awareness and syllable reading |
| Teaching session 1 | Reading task, dice game and lotto game  Syllables learned (set 1): “ba”, “bi”, “fa”, “fi”  Syllables learned (set 2): “bo”, “bu”, “fo”, “fu” | Reading task, dice game, matching game and lotto game  Letters learned (set 1): “b”, “f”, “a”, “i”  Letters learned (set 2): “b”, “f”, “o”, “u” |
| Teaching session 2 | Reading task, dice game and lotto game  Syllables learned (set 1): “so”, “su”, “to”, “tu”  Syllables learned (set 2): “sa”, “si”, “ta”, “ti” | Reading task, dice game, matching game and lotto game  Letters learned (set 1): “s”, “t”, “o”, “u”  Letters learned (set 2): “s”, “t”, “a”, “i” |
| Teaching session 3 | Reading task, dice game, lotto game and relay game  Syllables trained (set 1): “ba”, “bi”, “so”, “su”  Syllables trained (set 2): “bo”, “bu”, “sa”, “si” | Reading task, dice game, lotto game and relay game  Letters trained (set 1): “b”, “s”, “i”, “o”  Letters trained (set 2): “b”, “s”, “i”, “o” |
| Teaching session 4 | Reading task, dice game, lotto game and relay game  Syllables trained (set 1): “fa”, “fi”, “to”, “tu”  Syllables trained (set 2): “fo”, “fu”, “ta”, “ti” | Reading task, dice game, lotto game and relay game  Letters trained (set 1): “f”, “t”, “u”, “a”  Letters trained (set 2): “f”, “t”, “u”, “a” |
| Test at T2 | Phonemic awareness | Phonemic awareness |
| Coding-decoding session | Letters & Syllables (set 1): “b”, “f”, “t”, “s”, “a”, “i”, “o”, “u” and “ba”, “bi”, “fa”, “fi”, “so”, “su”, “to”, “tu”  Letters & Syllables (set 2): “b”, “f”, “t”, “s”, “a”, “i”, “o”, “u” and “bo”, “bu”, “fo”, “fu”, “sa”, “si”, “ta”, “ti” | Letters & Syllables (set 2) : “b”, “f”, “t”, “s”, “a”, “i”, “o”, “u” and “bo”, “bu”, “fo”, “fu”, “sa”, “si”, “ta”, “ti”  Letters & Syllables (set 1): “b”, “f”, “t”, “s”, “a”, “i”, “o”, “u” and “ba”, “bi”, “fa”, “fi”, “so”, “su”, “to”, “tu” |
| Tests at T3 | Letter knowledge, phonemic awareness and syllable reading | Letter knowledge, phonemic awareness and syllable reading |
| Test material | Syllables Set 1: “bac” /bak/, “bit” /bit/, “fat” /fat/, “fip” /fip/, “sol” /sol/, “suk” /syk/, “tol” /tol/, “tup” /typ/  Syllables Set 2: “boc” /bok/, “bul” /byl/, “fok” /fok/, “fut” /fyt/, “sap” /sap/, “sil” /sil/, “tal” /tal/, “tip” /tip/  Novel Syllables : “vip” /vip/, “vuc” /vuk/, “pat” /pat/, “pol” /pol/, “mot” /mot/, “mul” /myl/, “rap” /Rap/, “ric” /Rik/,  Letters: “b” /b/, “f” /f/, “t” /t/, “s” /s/, “a” /a/, “i” /i/, “o” /o/, “u” /y/ | |
| Teaching sessions material | Syllables Set 1: “ba” /ba/, “bi” /bi/, “fa” /fa/, “fi” /fi/, “so” /so/, “su” /sy/, “to” /to/, “tu” /ty/  Syllables Set 2: “bo” /bo/, “bu” /by/, “fo” /fo/, “fu” /fy/, “sa” /sa/, “si” /si/, “ta” /ta/, “ti” /ti/  Letters: “b” /b/, “f” /f/, “t” /t/, “s” /s/, “a” /a/, “i” /i/, “o” /o/, “u” /y | |
